# Tuning of Silver Content on the Antibacterial and Biological Properties of Poly(ɛ-caprolactone)/Biphasic Calcium Phosphate 3D-Scaffolds for Bone Tissue Engineering

**DOI:** 10.3390/polym15173618

**Published:** 2023-08-31

**Authors:** Francesca Menotti, Sara Scutera, Bartolomeo Coppola, Fabio Longo, Narcisa Mandras, Lorenza Cavallo, Sara Comini, Rosaria Sparti, Elisa Fiume, Anna Maria Cuffini, Giuliana Banche, Paola Palmero, Valeria Allizond

**Affiliations:** 1 Department of Public Health and Pediatrics, University of Torino, 10126 Turin, Italy; francesca.menotti@unito.it (F.M.); sara.scutera@unito.it (S.S.); f.longo@unito.it (F.L.); narcisa.mandras@unito.it (N.M.); lorenza.cavallo@unito.it (L.C.); rosaria.sparti@unito.it (R.S.); annamaria.cuffini@unito.it (A.M.C.); valeria.allizond@unito.it (V.A.); 2 Department of Applied Science and Technology, Politecnico di Torino, 10129 Turin, Italy; bartolomeo.coppola@polito.it (B.C.); paola.palmero@polito.it (P.P.)

**Keywords:** poly(ε-caprolactone)-based biomaterial, calcium phosphates, silver, *Staphylococcus aureus*, *S. epidermidis*, *Escherichia coli*, anti-adhesive/antibacterial properties, Saos-2 cells’ cell viability/proliferation

## Abstract

There is a growing interest in tissue engineering, in which biomaterials play a pivotal role in promoting bone regeneration. Furthermore, smart functionalization can provide biomaterials with the additional role of preventing orthopedic infections. Due to the growing microbial resistance to antimicrobials used to treat those infections, metal ions, such as silver, thanks to their known wide range of bactericidal properties, are believed to be promising additives in developing antibacterial biomaterials. In this work, novel poly(ε-caprolactone) (PCL)-based 3D scaffolds have been designed and developed, where the polymer matrix was modified with both silver (Ag), to supply antibacterial behavior, and calcium phosphates (biphasic calcium phosphate, BCP) particles to impart bioactive/bioresorbable properties. The microstructural analysis showed that constructs were characterized by square-shaped macropores, in line with the morphology and size of the templating salts used as pore formers. Degradation tests demonstrated the important role of calcium phosphates in improving PCL hydrophilicity, leading to a higher degradation degree for BCP/PCL composites compared to the neat polymer after 18 days of soaking. The appearance of an inhibition halo around the silver-functionalized PCL scaffolds for assayed microorganisms and a significant (*p* < 0.05) decrease in both adherent and planktonic bacteria demonstrate the Ag+ release from the 3D constructs. Furthermore, the PCL scaffolds enriched with the lowest silver percentages did not hamper the viability and proliferation of Saos-2 cells. A synergic combination of antimicrobial, osteoproliferative and biodegradable features provided to 3D scaffolds the required potential for bone tissue engineering, beside anti-microbial properties for reduction in prosthetic joints infections.

## 1. Introduction

Bone tissue diseases are a growing concern due to the aging population. In fact, adverse clinical conditions, attributable to trauma, pathologies, tumors, or previous surgical procedures, may affect the population, especially those over 50 [1]. While tissue regeneration is a natural process, certain clinical cases may benefit from tissue engineering to boost the production of new bone tissue. Self-regeneration is a multifaceted biological process that includes molecular signaling and many different types of cells. Osteoblasts, as bone synthesizing cells, produce the bone matrix, and, subsequently, the remodeling of new bone tissue occurs to finalize mature bone [2,3]. However, these physiological healing processes decrease in older people; therefore, tissue engineering can offer a new approach for bone restoration and the improvement of medical procedures [1,4].

Engineered bone graft substitutes are designed to restore impaired bone tissue and its mechanical properties, allowing, at the same time, natural bone regeneration and healing progression. Deficient tissue can be repaired and substituted by a three-dimensional (3D) porous construct known as a “scaffold”, which has been identified as an innovative therapeutic answer for cavity filling in bone pathological alterations or for the healing of fractured bone [1]. Such bone biocompatible 3D scaffolds may be obtained from natural or synthetic sources. The latter comprise polymers such as polycaprolactone (PCL), a nontoxic aliphatic polyester with FDA-approved clinical applicability thanks to its biodegradability and biocompatibility properties [5,6]. In medical applications, PCL is investigated for wound dressing [7,8], fixation implants [9], drug delivery [10], or in bone-tissue engineering for the manufacturing of long-term implantable devices [3,5,6,11,12].

In order to better match the compositional, physical, and mechanical properties of natural bone, synthetic polymers can be added with ceramic particles, mainly hydroxyapatite (HA), α/β-tricalcium phosphate (α/β-TCP) or biphasic calcium phosphate (BCP, a mixture of HA/β-TCP), known as the mineral components of the natural bone [1,6,13,14]. The incorporation of calcium phosphates (CaPs), as the bioactive component, into PCL provides the 3D scaffold with the required multi-functionalities, merging mechanical features, bioactivity, biodegradability, and the ability to stimulate, promote, and induce osteogenic cell differentiation [3,15].

Furthermore, it is pivotal that these 3D scaffolds can be additionally tuned to let the controlled—fast or prolonged—release of antibacterial agents, allowing the development of novel personalized tissue engineering applications with the ability to act as a targeted delivery system, achieving a high local concentration of loaded molecules with a dose-dependent effect [13,16]. PCL itself could be the cargo of antimicrobial substances such as copper, silver, zinc oxide, or graphene [15,17]. Notably, silver displays a wide spectrum of antimicrobial activity against different pathogens, including those resistant to various antibiotics. In addition, due to its low toxicity to mammalian cells [8,16], the use of silver in orthopedics appears to be a strategic choice.

In one of our previous studies [11], with the aim of developing antibacterial and anti-biofilm biomaterials for tissue regeneration, we focused on the design and manufacturing of BCP/PCL composites functionalized with silver at 1.67wt% with respect to the polymer matrix. These 3D scaffolds showed a highly porous structure, suitable mechanical properties, and relevant antibacterial/anti-adhesive activity against *Staphylococcus aureus* as well. On the other hand, despite the fact that the silver content was relatively low, they displayed cytotoxic behavior towards eukaryotic cells, specifically human osteoblasts.

For these reasons, in the present research, the silver content in the BCP/PCL porous architecture was tuned in the range ~0.8–1.2%, featuring a 3D scaffold characterized by both antibacterial/anti-adhesive and osteogenic properties. This research presents a comparative study of the antibacterial properties of the 3D scaffolds made of BCP/PCL with the addition of varying silver content against three typical human pathogens—*S. aureus*, *S. epidermidis,* and *Escherichia coli*—involved in orthopedic infections.

## 2. Materials and Methods

### 2.1. BCP/PCL-Based Scaffold Preparation and Characterization

Poly(ε-caprolactone) (PCL, Merck KGaA, Milan, Italy) pellets were solubilized in acetone (20 wt%) at 40 °C for 24 h. In order to fabricate 3D porous structures, two types of inorganic salts, with sizes in the range 125–355 µm, were used as pore formers, precisely NaCl and NaNO_3_ (Sigma Aldrich, St. Louis, MO, USA, >99.5% purity). Salt granules were mixed with the solubilized polymer (NaCl or NaNO_3_:PCL weight ratio 90:10); once homogenized, the suspension was cast into cylindrical plastic molds (∅ = 20 mm, h = 10 mm). Once dried and demolded, samples were soaked in deionized water for 4 days, renewing the water each day, to solubilize the salt and generate the required porosity. To fabricate the porous poly(ε-caprolactone)/biphasic calcium phosphate (BCP/PCL) composites, first HA (Captal S BM192, Plasma Biotal Limited, Buxton, UK) and β-TCP (Captal R, Plasma Biotal Limited) powders were mixed under dry conditions in a 70:30 weight ratio to provide the BCP mixture. BCP composite powder was then added to acetone and stirred for 12 h, to which PCL was finally added (BCP:PCL weight ratio, 40:60). Further details on the experimental process can be found in Comini et al. 2021 [11]. Finally, for the (Ag)-doped materials, a variable concentration of silver nitrate (AgNO_3_, Grade AR, Sigma Aldrich) was mixed with acetone until complete dissolution. In particular, the 1 wt% or 1.2 wt%, and the 0.79 wt% or 1 wt% (as respect to PCL) were introduced for the NaCl or NaNO_3_ 3D-scaffolds, respectively. These silver concentrations to be added to the polymer were determined by preliminary tests aimed at providing an antibacterial action against the three different microorganisms while preserving the viability of the human osteosarcoma (SaoS)-2 cells. These preliminary results were obtained by testing—using the micro-dilution assays [18]—the direct effect of silver on both bacteria and eukaryotic cells.

Details of all the obtained specimens are reported in Table 1.

The microstructural characterization was carried out by field emission scanning electron microscopy (FESEM, Zeiss Supra 40, Jena, Germany).

The phase composition was investigated with X-ray diffraction (XDR, Philips PW 1710, Eindhoren, The Netherlands) analysis. In particular, the relative ratio between the HA and β-TCP phases was determined by the following relationship [19]:

(1) $$\%HA=\frac{I_{100}(HA)}{\begin{array}{c} I_{100}\left(HA\right)+1_{100}\left(TCP\right) \end{array}}\times 100$$

where I_100_ (HA) and I_100_ (TCP) denote the relative peak intensities of the hydroxyapatite and β-TCP phases, respectively.

Simultaneous Thermogravimetry-Differential thermal analysis (TG-DTA, LabSys evo machine, Setaram, Caluire, France) was performed on both PCL and BCP/PCL samples to assess the influence of the ceramic filler on the thermal behavior of the polymer.

### 2.2. PCL-Based and BCP/PCL-Based 3D Scaffold Biodegradability Test

The biodegradability tests were performed as previously reported in detail [12]. Briefly, the BCP/PCL-based construct —prepared with different silver concentrations—was immersed in Dulbecco’s modified eagle medium (DMEM; Merck KGaA) solution [12,20,21,22] at 37 °C during 3, 6, 12, and 18 days of incubation. After each soaking time, the scaffolds were removed from the DMEM and dried to reach a constant mass before weighing. The weight loss values of the pure PCL and composite BCP/PCL samples were obtained following this formula:

(2) $$weight\;loss\left(\%\right)=\frac{m_{0}-m_{x}}{m_{0}}\times 100$$

where

m_0_ = initial mass of the sample
m_x_= mass of the dried sample after immersion at time x

### 2.3. Cell Viability Assays by Direct-Contact Assay

The in vitro cytotoxicity test was performed, as previously detailed [11,12], on human Saos-2 (American Type Culture Collection^®^, ATCC^®^, HTB-85, Manassas, Virginia, VA, USA), an osteosarcoma cell line characterized by an osteoblastic phenotype. These eukaryotic cells were cultured in DMEM high in glucose (Merck KGaA) with phenol red (plus sodium bicarbonate, 10% fetal bovine serum, and 1% penicillin–streptomycin), and incubated at 37 °C in a 5% CO_2_ atmosphere.

The sterile BCP/PCL 3D scaffolds were cut into cylinders of 5 mm diameter and 5 mm height and put in 96-well plates; thereafter, their surface was covered by 2 × 10^4^ Saos-2 cells and incubated in culture medium for different incubation times, specifically 0, 3, 6, and 12 days. All the BCP/PCL-based samples, with or without silver, were assayed in triplicate, and the medium was replaced every 2–3 days. At each time point, the cell viability was determined by the 3-(4,5-Dimethylthiazol-2-yl)-2,5-Diphenyltetrazolium Bromide (MTT) assay (Merck KGaA), and the optical density (OD) was measured at 570 nm using a microplate reader (VICTOR3TM, PerkinElmer, Waltham, MA, USA). The results were reported as OD values, subtracting the OD of the PCL or BCP/PCL-based scaffolds without Saos-2 cells from the OD of the PCL or BCP/PCL-based 3D scaffolds with Saos-2 cells. Differences among the obtained OD values on the various constructs with and without silver were determined and statistically analyzed.

### 2.4. In Vitro Antibacterial Assays

The in vitro antibacterial assays were performed by testing different pathogens involved in orthopedic infections [23,24,25], specifically *S. aureus* (ATCC^®^ 29213), *S. epidermidis* (ATCC^®^ 35984), and *E. coli* (ATCC^®^ 25922). To investigate the activity of silver blending to PCL- or BCP/PCL-based 3D constructs, the inhibition halo (manual v 9.0; https://www.eucast.org/ast_of_bacteria/disk_diffusion_methodology, accessed on 30 July 2023) and the bacterial adhesion experiments were conducted on the three bacterial strains as recently reported in our research [11,12].

Briefly, for the inhibition halo test, 0.5 McFarland (1–2 × 10^8^ colony-forming units, CFU/mL) suspensions of each bacterium were uniformly spread on Mueller Hinton Agar (MHA, Becton Dickinson and Company, BD, Franklin Lakes, NJ, USA), then sterile both PCL- or BCP/PCL-pure scaffolds and the correspondingly silver-added ones were placed on agar. The silver release from functionalized specimens and its effect on bacterial development were evaluated after an incubation for 24 h at 35 ± 2 °C by measuring (mm) the inhibition halo [11,12].

Whereas, for the microbial adhesion test, as previously detailed [11,12], the bacteria were cultured for 18–24 h at 35 ± 2 °C in Mueller Hinton Broth (MHB, BD), then centrifuged and diluted in MHB to obtain a 10^4^ CFU/mL inoculum. The sterile PCL- or BCP/PCL-based 3D constructs, with and without different silver contents, were surrounded by 7 mL of bacterial inoculum in a 6-well culture plate and incubated at 35 ± 2 °C by shaking for 24 h to permit in vitro bacterial bonding to the biomaterials. After incubation, the constructs were subjected to sonication for 30 min at room temperature in 10 mL of NaCl 0.9% solution (Bieffe Medital S.p.A., Grosotto, Italy) to detach bacteria, which were strongly bound to the samples themselves and quantified by a plating count on MHA. Planktonic bacteria were also counted as CFU/mL. The adhesion tests were conducted in triple for each 3D construct type and performed at least three times.

### 2.5. Statistical Analysis

The GraphPad Prism 9 software (San Diego, CA, USA) was employed to analyze the morphological parameters, the weight loss percentages, the microbiological (CFU/mL), and the MTT (OD, 570 nm) data by descriptive statistics (means and standard error of the means). An unpaired Student’s *t*-test was used to find significant differences (*p <* 0.05) between the various tested samples.

## 3. Results and Discussion

The present study was aimed at designing and developing 3D scaffolds based on pure PCL or BCP/PCL functionalized biomaterials for orthopedic bone tissue engineering that would, in parallel, ensure bone regenerative potential through the presence of BCP and an antimicrobial feature by adding AgNO_3_, released as Ag^+^. The available literature pertains to PCL as fibers or printed constructs, but only when added with metal ions (i.e., silver, copper, zinc, etc.) [5,13,14,15,16,26,27], whereas no existing research has studied the combined use of calcium phosphates and silver, especially on 3D constructs fabricated by the salt leaching/polymer casting method.

### 3.1. Characterization of PCL- and BCP/PCL-Based Biomaterials

The dimensions of all the PCL- and BCP/PCL-based biomaterials are reported in Table 2. In brief, the 3D scaffolds exhibited a morphological cylinder-shaped geometry with comparable dimensions, specifically diameter (mm) and height (mm). Composite samples (BCP/PCL) presented a slightly larger diameter compared to pure PCL, indicating a lower shrinkage during drying of the former specimens, imputable to the constraining effect of the ceramic particles. Density values showed almost superimposable values among the same type of samples but obtained with different salts (NaCl or NaNO_3_). Besides the negligible role of salts on density, these results highlight the high reproducibility of the process employed.

In addition, considering the theoretical density of neat PCL (1.145 g/cm^3^), the density of pure PCL specimens (0.126 ± 0.003 g/cm^3^ and 0.127 ± 0.003 g/cm^3^ for the 3D scaffolds pored with NaCl or NaNO_3_, respectively) provided a total porosity of ~89%, in fair agreement with the expected nominal porosity of 90%. When the BCP was added to obtain the BCP/PCL composite constructs, the density increased to about 0.22 g/cm^3^. This augmentation can be explained by considering the theoretical density of the ceramic phases (3.16 g/cm^3^ for HA and 3.07 g/cm^3^ for β-TCP), which provided a nominal density for the BCP/PCL composite of 1.53 g/cm^3^ (as determined by the rule of mixture). Therefore, in this case, the overall porosity was ~86%, just slightly lower than the nominal 90% volume.

Notably, the addition of the different percentages of silver to the 3D scaffolds, either pure PCL or BCP/PCL, did not significantly modify neither the cylindrical geometry nor the dimensions, while a slight increase in density was observed (Table 2).

**Figure 1:**
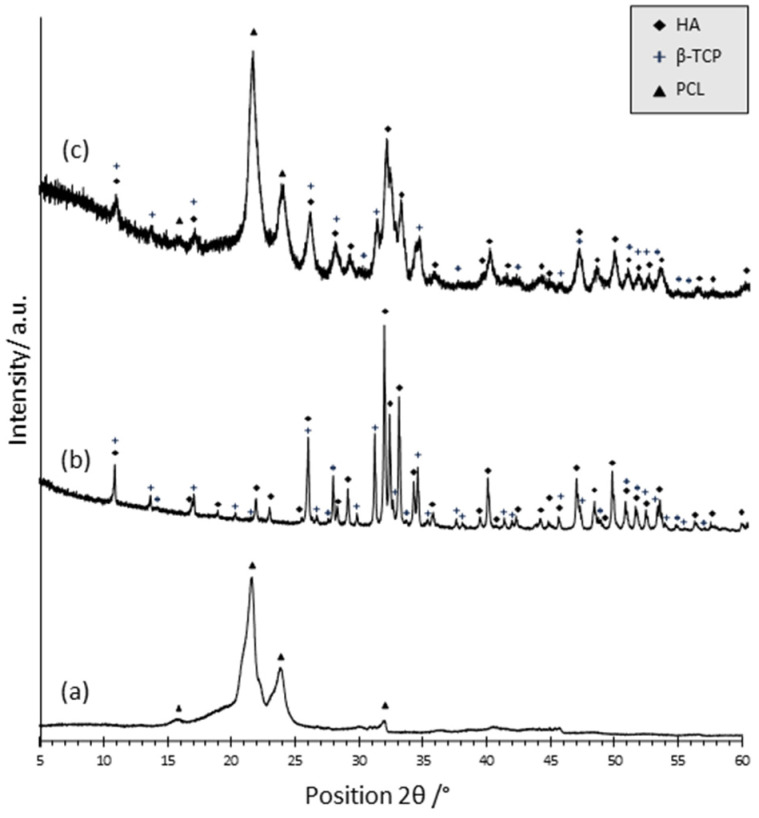
XRD patterns of neat PCL (**a**), of the HA:β-TCP composite powder (**b**), and of the composite BCP/PCL scaffold (**c**).

In Figure 1, the XRD patterns of neat PCL (a) and of BCP composite powder (b)—both used as references—and the pattern related to BCP/PCL composite scaffold (c) are presented. Concerning the neat polymer (a), the presence of two sharp peaks at 21.65 (°2θ) and 23.92 (°2θ), corresponding respectively to the (110) and (200) planes, denotes the presence of polycaprolactone with a semi-crystalline structure, in agreement with literature data [28,29]. In (b), the peaks of the HA and β-TCP phases were recognized and indexed through the JCPD files n. 00-009-0432 and 00-009-0169, respectively. By applying Equation (1), the HA: β-TCP weight ratio was 68:32. The fair agreement between the experimental and nominal ratios (70:30) strengthens the use of the above equation for the semi-quantification of the calcium phosphate phase’s ratio. In (c), the PCL main signals have been determined, along with all the most intense peaks of the HA and β-TCP phases. These results suggest a negligible role for calcium phosphate particles in modifying the crystallinity of the polymer matrix. Equation (1) provides that the HA and β-TCP phases are present in the polymer matrix with a weight ratio of 72:28, still in very good agreement with the nominal ratio.

In addition, in order to quantify the PCL:BCP ratio, DTA-TG analysis was carried out on the composite scaffold. After thermal decomposition at 650 °C, a residual mass equal to 43% was determined, corresponding to the calcium phosphate particles, in agreement with the nominal content (40 wt% with respect to PCL).

Joint results concerning the calcium phosphate content in the polymer (by DTA-TG) and on the HA:β-TCP ratio (by XRD) clearly demonstrate that the long water soaking time of the samples (4 days), necessary to leach out the NaCl and NaNO_3_ salts, did not induce any significant or preferential dissolution of the calcium phosphate phases. Although the above results were determined on samples prepared by using NaCl as a pore former, similar results were determined with NaNO_3_ and therefore were not repeated here.

**Figure 2:**
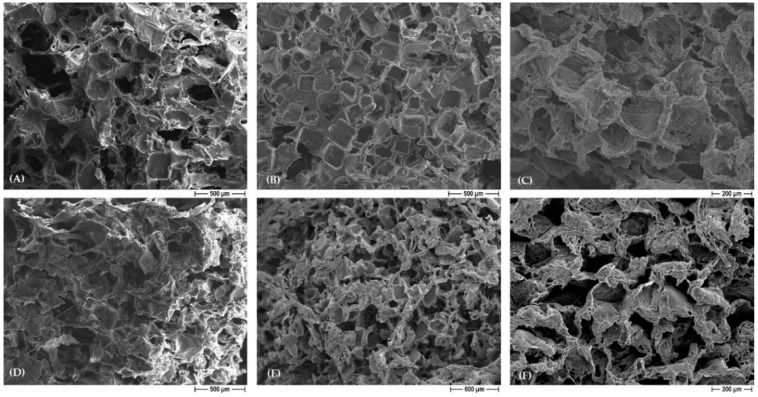
Representative FESEM micrographs of neat PCL scaffolds (**A**,**D**) and silver-added PCL (**B**,**C**,**E**,**F**) obtained by using NaCl (**A**–**C**) and NaNO_3_ (**D**–**F**) as pore formers. For silver-added PCL, lower (**B**,**E**) and higher (**C**,**F**) magnification images are depicted.

FESEM micrographs of neat PCL scaffolds as well as silver-added PCL and BCP/PCL composite constructs are illustrated in the following: In Figure 2, the morphology of the PCL scaffolds pored with NaCl (A) and NaNO_3_ (D) salts is compared. While in the former case the pores are characterized by well-defined geometrical shapes due to regular-shaped NaCl granules [11,12], a less regular pore shape can be recognized in NaNO_3_-derived materials. This difference in pore morphology is maintained in the micrographs of the silver-added PCL scaffolds, where squared pores characterized the NaCl-based sample (B,C), contrary to the NaNO_3_-one (E,F). In spite of this, all the structures were characterized by a very high porosity and were open and interconnected (as evidenced by the higher magnification images, C and F), which is extremely required in bone tissue engineering. Notably, the addition of silver did not alter the pore morphology or the microstructures, as the same features can be observed in neat and silver-added PCL specimens.

**Figure 3:**
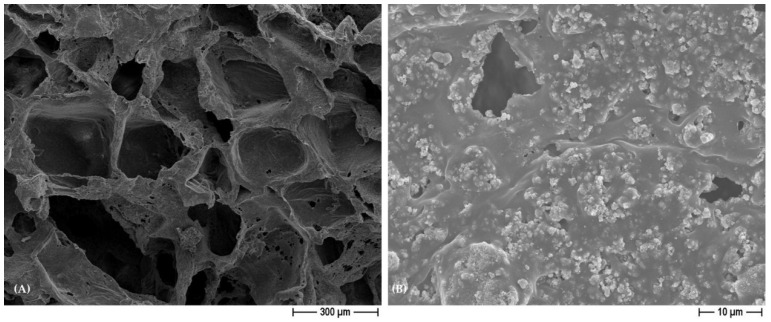
Representative FESEM micrographs of BCP/PCL (NaCl) scaffolds at lower (**A**) and higher (**B**) magnification.

Figure 3 shows the FESEM micrograph of BCP/PCL scaffolds obtained by using NaCl as a pore former, as indicated by the geometrically shaped pores. The lower magnification image (A) shows again the high porosity degree and the pore interconnection by means of tine pores in the cell walls; the higher magnification one (B) shows the homogeneous dispersion of the HA/β-TCP particles, well embedded into the polymer matrix. Similar microstructural features were observed in the NaNO_3_-based samples and are thus not depicted here.

### 3.2. PCL- and BCP/PCL-Based 3D Scaffold Biodegradability Degree

**Figure 4:**
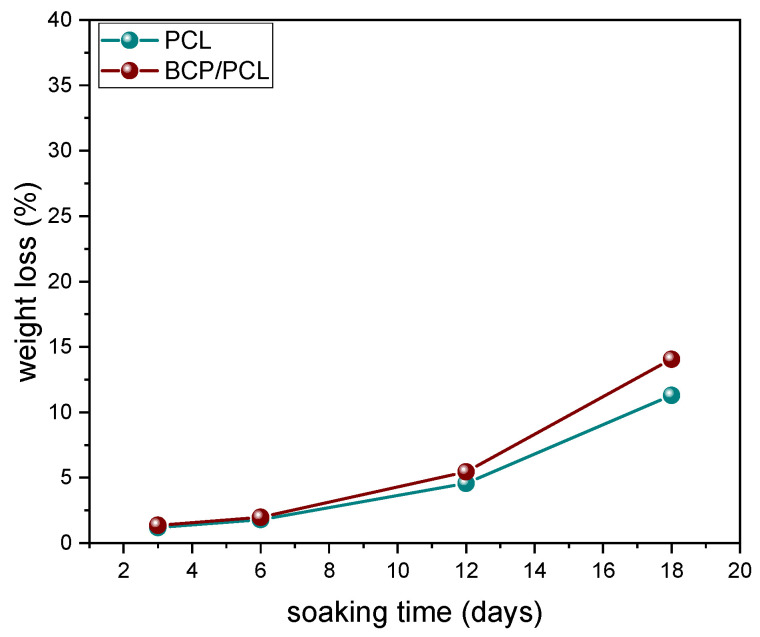
Weight loss of PCL and BCP/PCL scaffolds (by using NaCl as pore former salt) at different time points (3, 6, 12, and 18 days) after the immersion in DMEM.

In Figure 4, the weight loss of the 3D scaffold as a function of the immersion time in DMEM is depicted.

For both PCL and BCP/PCL scaffolds, a gradual increase in mass loss was determined, with close values for the two types of samples up to 12 days (4.6% and 5.5%, respectively). After 18 days, a more significant mass loss was determined for the composite sample, reaching 14% of the weight loss compared to the neat polymer scaffold (11%).

These results are in poor agreement with literature data since, generally, a lower degradation degree for PCL-based materials is reported [30,31]. However, a clear comparison of results is difficult due to the various experimental conditions used to test the materials (such as soaking medium, time and temperature, degradation mechanism, etc.), as well as the different material features (such as porosity amount and size, crystallinity, thickness of the samples, etc.). With specific reference to biodegradability tests performed in DMEM, Lu et al. (2012) fabricated scaffolds by injecting the melt polymer or melt PCL added with β-TCP particles into a soluble porous mold [32]. The authors reported a weight loss of just 0.35% after 2 weeks of immersion, which increased to ~3% and 4% after 4 and 6 weeks, respectively. These values increased significantly for 10% and 20% β-TCP-containing materials, showing 2.4% and 2.8% weight loss, respectively, after 2 weeks of immersion. A very low weight loss (0.7%) after 2 weeks of immersion of PCL scaffold, obtained by fused deposition modeling technique, was also reported by Hedayati et al. (2022) too [33]. Muhammad et al. (2012) compared the biodegradability of a commercial PCL scaffold with a self-crossing PCL-derived polymer, the polycaprolactone trifumarate, obtained by a salt-leaching method, similarly to our work but using a lower polymer: NaCl ratio of 1:1 [34]. While the former still presented a moderate weight loss after 2 weeks (~2.5%) but was comparable to the values achieved in this work after 12 days, the modified PCL achieved values close to 20%. Finally, Janarthanan et al. (2019) reported weight loss of ~8% and 10% for porous PCL and porous PCL/α-TCP, obtained by a solvent casting/salt leaching method similar to our process after 14 days of immersion, in good agreement with the present results [35]. The highly porous structure, characterized by open and well-interconnected pores and thin struts within the cells, is probably responsible for this high degradation degree and rate. A further explanation, when comparing the current results with tests carried out in other media, can be ascribed to the better wettability of PCL by DMEM than other mediums like physiological buffer solution (PBS), as determined by Musciacchio et al. (2022) [36].

**Figure 5:**
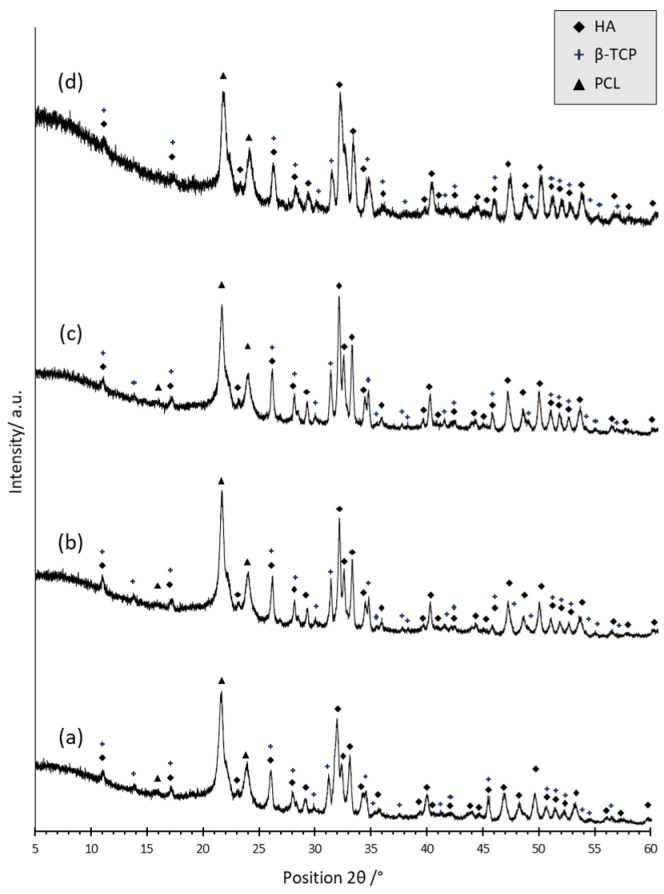
XRD patterns of BCP/PCL scaffolds after soaking for 3 (**a**), 6 (**b**), 12 (**c**), and 18 (**d**) days in DMEM.

In this work, to rule out a selective release of calcium phosphate particles during soaking in DMEM, XRD analyses were performed on BCP/PCL samples before and after each incubation time (Figure 5).

Samples soaked in DMEM for different times showed very similar diffraction patterns in terms of the intensity of the signals associated with PCL, HA, and β-TCP phases. By applying Equation (1), the HA: β-TCP ratio was in fact 71.5, 70, 70, and 73 in samples incubated for 2, 6, 12, and 18 days, respectively, showing a very good match with the nominal ratio. These results rule out any significant dissolution of the ceramic particles into the incubation fluid; on the other hand, calcium phosphate particles can play a role in improving the hydrophilicity of PCL, thus explaining the highest degradation of BCP/PCL compared to the neat polymer after 18 days of soaking.

### 3.3. In Vitro Saos-2 Cell Viability/Proliferation Assay

Here, we evaluated Saos-2 cells since these eukaryotic cells are a useful in vitro model of typical osteoblast behavior and represent a mature osteoblast phenotype with high alkaline phosphatase activity and osteocalcin expression at similar levels as human primary osteoblasts [37,38,39].

In our previous paper, we demonstrated that the silver concentration of ~1.67%, even if it showed good efficacy against *S. aureus*, unfortunately impaired Saos-2 cell viability and proliferation [11]. Thus, here we tuned the silver amount into the 3D scaffolds to reach both antibacterial and non-cytotoxic behavior. Specifically, regarding samples pored with NaCl, they were added with 1% and 1.2%, whereas those pored with NaNO_3_—for which we demonstrated a higher release of silver content into the medium [11]—were functionalized with 0.79% and 1%.

**Figure 6:**
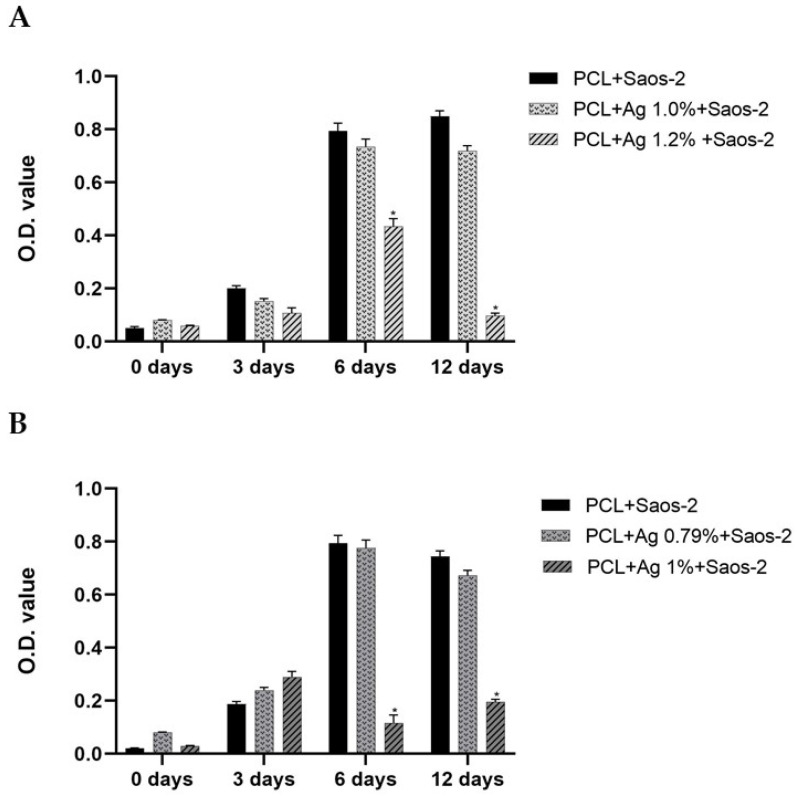
Cell viability (MTT analysis) of Saos-2 cells exposed to pure PCL-based biomaterial and to different concentrations of Ag-enriched PCL 3D scaffolds, fabricated with NaCl (**A**) or NaNO_3_ (**B**) salts, expressed as optical density (OD) value at 570 nm. Results are means ± standard error of the mean (SEM) of at least three independent experiments; * *p* < 0.001 unpaired *t*-test.

In Figure 6, the summary of all the results achieved by the MTT assays and expressed as optical density (OD 570 nm) concerning the viability and proliferation of Saos-2 cells in contact with the different specimens pored with NaCl (A) or NaNO_3_ (B) within 12 days of incubation, blended or not with silver, is reported.

Briefly, within 3 days of incubation, similar OD values were recorded for all the PCL-based 3D scaffolds, reporting no differences neither between controls and silver-functionalized specimens nor between the NaCl (A) and NaNO_3_ (B) salts used to form the pores in the samples. At 6 days of incubation, while the OD for the pure PCL and the PCL + 1% of silver for NaCl constructs, or 0.79% of silver for the NaNO_3_ ones, resulted in an additional increase in the OD values, the 3D scaffolds enriched with the higher silver concentrations—specifically 1.2% or 1% for NaCl and NaNO_3_, respectively—highlighted a significant (*p* < 0.001) decrease in the OD values with respect to pure PCL (Figure 6). Notably, after 12 days of incubations, a further significant (*p* < 0.001) reduction in cell viability was evident for the 1.2% (NaCl pored) and the 1% (NaNO_3_ pored) functionalized specimens, with respect to pure-PCL and to the ones added with the lower concentrations (1% or 0.79% for NaCl and NaNO_3_ pored constructs, respectively). An analogous but not cytotoxic pattern pertaining to the 3D scaffolds obtained with BCP/PCL and with BCP/PCL added with the lower (1% for NaCl and 0.79% NaNO_3_) silver concentrations was obtained, thus demonstrating that the functionalization with calcium phosphates did not impair eukaryotic cell viability.

Finally, for both pure-PCL and PCL enriched with the lower (1% or 0.79%) silver percentages, the preparation of the 3D scaffolds either with NaCl or with NaNO_3_ promoted Saos-2 proliferation within 6 days, determining a confluence state and no additional growth at 12 days (Figure 6).

In the attempt to screen the existing literature on the effect of PCL enriched with silver on sarcoma Saos-2 cells viability, we faced the fact that very poor articles on the issue are available; therefore, in the following part of the paper, we compared the direct action of silver towards these types of eukaryotic cells. Research by Ashe S. et al. (2016) demonstrated that the presence of AgNPs (0.005–0.25 mM) ameliorate Saos-2 cell parameters such as morphology and restores native protein structure [40]. The same authors [41] prepared composite hydrogels encompassing AgNPs and determined that, with respect to pure biomaterials, a slight reduction in Saos-2 cells was revealed, and the initial concentration of AgNPs was 1 mg/mL. More recently, Rodriguez-Contreras et al. (2023) demonstrated both adhesion and proliferation of Saos-2 cells on silver and gallium-modified titanium surfaces in a dose-dependent manner [25]. Additionally, in current studies, the direct effect of AgNPs (3–250 µg/mL or 0.3125–10 ppm) on SaoS-2 cells was tested, and a dose-dependent cytotoxicity was shown [42,43]. These data are in good agreement with those here obtained, since a decrease in eukaryotic cell viability was highlighted only when higher silver concentrations were used, whereas when they were exposed to lower ones, no cytotoxic effects were demonstrated. The silver concentrations that displayed a toxic or nontoxic behaviour towards Saos-2 cells are very close; thus, we can speculate that the blending of silver into the PCL- and BCP/PCL-based biomaterials allowed a tailored amount that was consequently specifically released as Ag^+^, demonstrating a cause/effect association between ions and cytotoxicity on eukaryotic cells [25,44].

In parallel, we further confirmed that the PCL polymer alone or blended with CaPs did not impair Saos-2 cell viability or proliferation [17,45,46].

### 3.4. Antibacterial Assays

A growing body of evidence indicates that the most common pathogens recovered from prosthetic joint infections (PJIs) in different clinical settings are Gram-positive bacteria belonging to the *Staphylococcus* species, mainly *S. aureus* and *S. epidermidis* [47,48]. Thereafter, other microorganisms, such as *Enterobacteriaceae* (i.e., *E. coli* or *Kleblsiella pneumoniae*), can be the causative bacterial species [48,49]. These bacteria can worsen the infectious process by both being resistant to the antimicrobial treatment and by producing a well-established biofilm [47,50,51].

**Figure 7:**
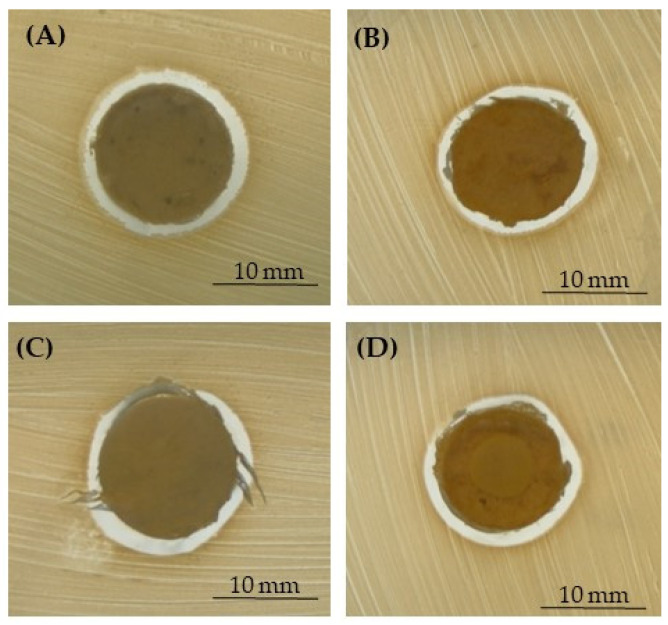
Representative images of the inhibition halo assay against *S. aureus* in the presence of the PCL-based samples, pored with NaCl, enriched with 1% (**A**) or 1.2% (**B**) of silver, or those pored with NaNO_3_, enriched with 0.79% (**C**) or 1% (**D**) of silver.

**Figure 8:**
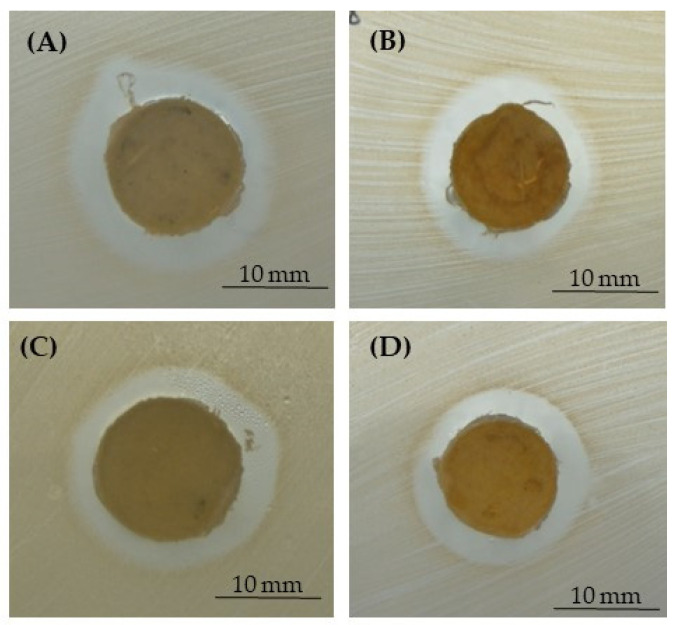
Representative images of the inhibition halo assay against *S. epidermidis* in the presence of the PCL-based samples, pored with NaCl, enriched with 1% (**A**) or 1.2% (**B**) of silver, or those pored with NaNO_3_, enriched with 0.79% (**C**) or 1% (**D**) of silver.

**Figure 9:**
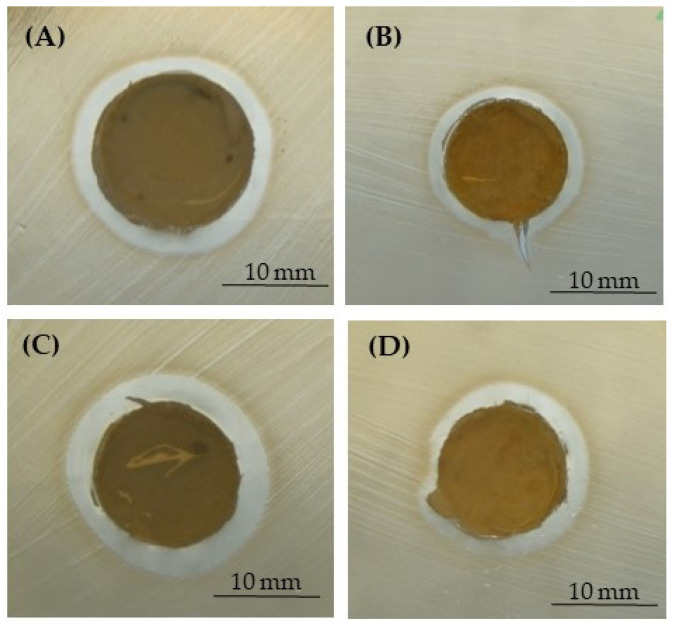
Representative images of the inhibition halo assay against *E. coli* in the presence of the PCL-based samples, pored with NaCl, enriched with 1% (**A**) or 1.2% (**B**) of silver, or those pored with NaNO_3_, enriched with 0.79% (**C**) or 1% (**D**) of silver.

For these reasons, three different pathogens—*S. aureus*, *S. epidermidis,* and *E. coli*—as demonstrative microorganisms involved in PJIs were used in the present research. The microbiological results were obtained by performing the inhibition halo (Figure 7, Figure 8 and Figure 9 and Table 3) and the bacterial adhesion experiments (Table 4 and Table 5). Even if only the lower concentration of silver (1% for NaCl and 0.79% for NaNO_3_) did not display a reduction in Saos-2 cell viability and proliferation, being non-toxic for eukaryotic cells, all the microbiological assays were performed by using all the silver concentrations added to the PCL-based biomaterials. In fact, literature research also reported that close concentrations of silver are not toxic for eukaryotic cells but exert antibacterial activity as well [24,25,27,42].

In Figure 7, Figure 8 and Figure 9, representative images of the inhibition halo assay are depicted, and in Table 3, the average diameter (mm) of the inhibition growth around the 3D scaffolds is reported. These data established that the silver was released from the PCL- and BCP/PCL-based Ag-added samples and that it acted towards the three microorganisms tested, even if a more pronounced activity was revealed against *S. epidermidis* with respect to both *S. aureus* and *E. coli*. No inhibition halo was obtained around the pure-PCL and -BCP/PCL constructs, confirming that no antibacterial action is displayed by the polymer alone or by the polymer functionalized by CaPs. These results are in line with those of other researchers [13,16,52]. In a recently published article, the authors demonstrated that a growth-inhibition zone was generated around titanium samples doped with silver and gallium against both Gram-positive (*S. aureus* and *S. epidermidis*) and Gram-negative (*E. coli* and *Pseudomonas aeruginosa*) bacteria [25]. Additionally, a similar wide area of inhibition was obtained by testing *S. aureus* halo around poly-lactic-co-glycolic-acid/PCL scaffolds enriched with AgNPs [53] or by assaying *S. aureus* and *E. coli* halo around scaffolds containing silver-doped HA [27].

The microbiological results (expressed as log_10_ CFU/mL) of the adhered bacteria—precisely *S. aureus*, *S. epidermidis,* and *E. coli*—to the different PCL- and BCP/PCL-based biomaterials, produced with either NaCl or NaNO_3_ salts, after 24 h of incubation are presented in Table 4.

Briefly, the three bacteria adhered to the pure PCL and BCP/PCL-based 3D scaffolds, used as controls, with different loads of 10^9^ CFU/mL, 10^7^ CFU/mL, and 10^8^ CFU/mL, for *S. aureus*, *S. epidermidis,* and *E. coli*, respectively. Whereas, with respect to the controls, the silver-blend specimens were able to significantly (*p* < 0.001) reduce their bacterial adhesion, highlighting values at 10^3^ CFU/mL for *S. aureus* and 10^2^ CFU/mL for both *S. epidermidis* and *E. coli*. As expected, no differences between the two silver concentrations (1% and 1.2% for NaCl and 0.79% and 1% for NaNO_3_) were revealed, neither for the Gram-positive nor for the Gram-negative bacteria (Table 4), suggesting that despite the variable silver amount in the 3D scaffolds, a reduction in the bacterial adhesion occurred anyway.

In Table 5, the growth of the three different microorganisms in the broths being in contact for 24 h with the specimens either controls—PCL and BCP/PCL—or silver-added ones, determined by the planktonic count as log_10_ CFU/mL, is shown.

These results confirmed that the silver was released by the enriched samples into the bacterial medium and exerted an antibacterial effect on their growth. In fact, a significant (*p <* 0.001) reduction in the planktonic *S. aureus*, *S. epidermidis,* and *E. coli* was detected, represented by a load of 10^5^ CFU/mL, 10^4^ CFU/mL, and 10^3^ CFU/mL, respectively. Whereas, in agreement with other literature results, no innate antibacterial activity by the polymer alone or functionalized with CaPs was highlighted [6,16,27,52].

**Figure 10:**
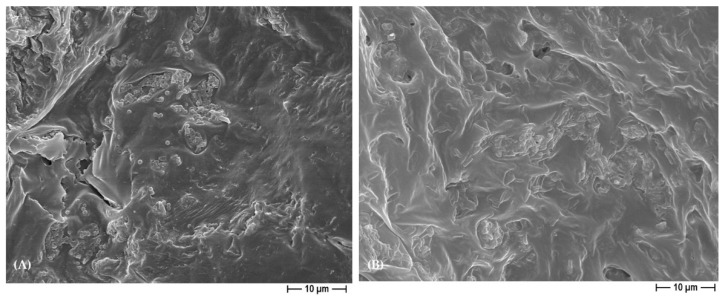
Representative FESEM micrograph of the control biomaterial, specifically BCP/PCL, non-sonicated in the presence of the Gram-positive (**A**) or the Gram-negative (**B**) bacteria, obtained by using NaCl salt as a template, at 5000× magnification, revealing a well-structured biofilm.

**Figure 11:**
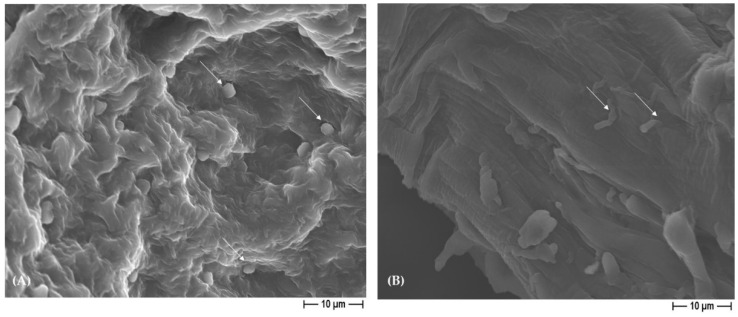
Representative FESEM micrographs reporting bacterial morphological modifications: *S. epidermidis* (**A**) has an oval-shaped morphology (white arrows) and *E. coli* (**B**) has an elongated morphology (white arrows) on PCL-based scaffolds added with 1% silver, obtained by using NaCl salt as a pore former (2000× magnification).

Thus, the silver presence in the 3D scaffolds revealed an anti-adhesive and antibacterial (Table 4 and Table 5) action, and additionally to that, an anti-biofilm activity. In fact, it has to be highlighted that a well-structured biofilm was noted in the control materials for both the Gram-positive and the Gram-negative bacteria (Figure 10), whereas in the silver-functionalized PCL and BCP/PCL-biomaterials, only a few microorganisms were observed, and notably, they were altered in their usual morphology (Figure 11). Actually, the staphylococci lost their spherical shape, and *E. coli* displayed a more elongated-fusiform morphology; this effect was due to the direct effect of silver on the bacterial external structures. These data are in line with those of other scientific works that have proposed a destabilization of the biofilm exerted by silver [25,54]. Here, we can speculate that the silver presence is able to inhibit the biofilm’s production rather than its dislocation.

All together, the microbiological results confirmed the anti-adhesive and anti-biofilm properties of the 3D scaffolds functionalized with both silver concentrations and, in parallel, antibacterial growth as well. In a study, the antimicrobial action of PCL/clay mineral vermiculite films doped or not with zinc was assayed, and the results demonstrated an antibacterial effect only against *E. coli* but not against *S. aureus* [5]. In addition, electrospun scaffolds containing silver-doped HA demonstrated a great antibacterial effect on *S. aureus* and *E. coli* growth over time [27]. Qian Y. et al. (2019) produced PLGA/PCL electrospun scaffolds, including AgNPs, and highlighted an inhibition in *S. aureus* growth [16]. Florea D.A. et al. (2022) evaluated the antibacterial performance of different coatings on magnesium phosphate-containing silver nanoparticles and demonstrated their strong anti-*S. aureus* and -*Ps. aeruginosa* efficiency [14].

## 4. Conclusions

In natural conditions, during an adult’s lifetime, bone regeneration and healing are well-orchestrated processes that involve different eukaryotic cells. However, the bone tissue remodeling due to different pathological events, such as bone fractures, tumors, skeletal abnormalities, and elderly diseases, requires a guiding substitute able to allow osteogenic cell proliferation and colonization. Notably, a relevant issue in the surgical implantation of a bone-tissue scaffold is the occurrence of infections that cause a shortcoming in the patient’s quality of life. These infections might be exacerbated by the presence of antimicrobial drug-resistant and biofilm-producing bacteria, too. Recent research has tried to overcome these problems by adding metal ions instead of antibiotics to scaffolds designed for bone tissue engineering. In this context, the present study demonstrated that the 3D PCL-based constructs functionalized with calcium phosphates showed a highly interconnected porosity and, in parallel, revealed a good degradation behavior able to promote eukaryotic cell colonization. Moreover, the tuning of the silver content into the novel BCP/PCL-based construct of about 1% revealed sustained anti-adhesive, antibacterial, and anti-biofilm activity—towards a broad spectrum of microbial species—without affecting osteoblast viability and proliferation. Further studies with human primary osteoblasts aimed at deepening their integration and differentiation when in contact with these new scaffolds will be investigated to promote bone tissue regeneration. The use of the appropriate concentration of silver displays many advantages over conventional antibiotics, mainly the targeted release and minimizing the problem of antibiotic resistance by bacteria. In fact, silver killing activity against microorganisms is complex and involves its action on various external layers of the bacterial cell and on proteins, DNA, and other inner structures; thus, the risk of selecting resistance is low. Hence, the here designed and studied 3D scaffolds based on PCL blended with calcium phosphates and blended with silver for bone tissue engineering are an effective strategy to be applied to improve the healing of such important human tissue.

## Figures and Tables

**Table 1:** 3D PCL-based constructs obtained for the present research.

Types of 3D Scaffolds	Salt Used as Pore Former	Composition of the 3D Scaffolds
pure PCL	NaCl	poly(ε-caprolactone)
pure BCP/PCL	NaCl	biphasic calcium phosphates/poly(ε-caprolactone)
PCL + Ag 1%	NaCl	poly(ε-caprolactone) + 1% of silver
PCL + Ag 1.2%	NaCl	poly(ε-caprolactone) + 1.2% of silver
BCP/PCL + Ag 1%	NaCl	biphasic calcium phosphates/poly(ε-caprolactone) + 1% of silver
BCP/PCL + Ag 1.2%	NaCl	biphasic calcium phosphates/poly(ε-caprolactone) + 1.2% of silver
pure PCL	NaNO_3_	poly(ε-caprolactone)
pure BCP/PCL	NaNO_3_	biphasic calcium phosphates/poly(ε-caprolactone)
PCL + Ag 0.79%	NaNO_3_	poly(ε-caprolactone) + 0.79% of silver
PCL + Ag 1%	NaNO_3_	poly(ε-caprolactone) + 1% of silver
BCP/PCL + Ag 0.79%	NaNO_3_	biphasic calcium phosphates/poly(ε-caprolactone) + 0.79% of silver
BCP/PCL + Ag 1%	NaNO_3_	biphasic calcium phosphates/poly(ε-caprolactone) + 1% of silver

**Table 2:** Morphological characteristics (reported as mean ± standard error of the mean) of the pure PCL- or BCP/PCL-based 3D scaffolds, functionalized with low silver concentrations, and pored with NaCl (A) or NaNO_3_ (B).

	Morphological Parameters			Statistical Analysis
A	**Diameter (mm)**	**Height (mm)**	**Density (mg/mm^3^)**	**Student’s *t*-Test**
Scaffold Type				
PCL	18.31 ± 0.11	11.27 ± 0.16	0.126 ± 0.003	weight and density PCL vs. BCP/PCL *p* < 0.001
BCP/PCL	18.98 ± 0.10	10.80 ± 0.29	0.204 ± 0.005	
PCL + Ag 1%	18.31 ± 0.21	10.58 ± 0.49	0.133 ± 0.009	
PCL + Ag 1.2%	18.38 ± 0.22	11.18 ± 0.49	0.132 ± 0.005	
BCP/PCL + Ag 1%	18.73 ± 0.12	11.68 ± 0.13	0.213 ± 0.004	
BCP/PCL + Ag 1.2%	18.92 ± 0.13	12.15 ± 0.16	0.221 ± 0.003	
B				
PCL	18.10 ± 0.13	10.10 ± 0.39	0.127 ± 0.003	weight and density PCL vs. BCP/PCL *p* < 0.001
BCP/PCL	18.74 ± 0.10	9.87 ± 0.41	0.205 ± 0.005	
PCL + Ag 0.79%	18.61 ± 0.13	11.11 ± 0.65	0.133 ± 0.009	
PCL + Ag 1%	18.42 ± 0.22	11.81 ± 0.46	0.132 ± 0.005	
BCP/PCL + Ag 0.79%	18.63 ± 0.11	9.64 ± 0.25	0.213 ± 0.004	
BCP/PCL + Ag 1%	18.99 ± 0.07	11.25 ± 0.16	0.220 ± 0.003	

Abbreviations: PCL—poly(ε-caprolactone); BCP—biphasic calcium phosphates; Ag—silver.

**Table 3:** Average diameters (reported as mean ± standard error of the mean) of the inhibition halo around the pure-PCL- or the BCP/PCL-based scaffolds functionalized with low silver concentrations, pored with NaCl (A) or NaNO_3_ (B), towards the three assayed bacterial strains.

	Average Diameter ± SEM (mm)		
A	***S. aureus***	***S. epidermidis***	***E. coli***
Scaffold Type pored with NaCl			
PCL + Ag 1%	22.65 ± 0.16	27.41 ± 0.21	22.02 ± 0.36
PCL + Ag 1.2%	22.85 ± 0.32	30.82 ± 0.30	22.19 ± 0.13
BCP/PCL + Ag 1%	22.79 ± 0.24	28.13 ± 0.12	21.32 ± 0.16
BCP/PCL + Ag 1.2%	23.06 ± 0.11	31.65 ± 0.22	22.87 ± 0.41
B			
Scaffold Type pored with NaNO_3_			
PCL + Ag 0.79%	23.12 ± 0.20	27.06 ± 0.38	21.11 ± 0.13
PCL + Ag 1%	23.13 ± 0.31	29.76 ± 0.18	22.29 ± 0.23
BCP/PCL + Ag 0.79%	24.09 ± 0.50	27.89 ± 0.22	21.51 ± 0.30
BCP/PCL + Ag 1%	24.23 ± 0.12	30.03 ± 0.47	22.77 ± 0.21

**Table 4:** Number of adherent staphylococci and *E. coli* (log_10_ colony-forming units, CFU/mL) on the PCL and BCP/PCL 3D scaffolds, pure or functionalized with low silver concentrations and pored with NaCl (A) or NaNO_3_ (B), towards the three assayed bacterial strains.

	Number of Adhered Bacteria as log_10_ CFU/mL (Means ± Standard Error of the Means)			Statistical Analysis
A	***S. aureus***	***S. epidermidis***	***E. coli***	**Student’s *t*-Test**
Scaffold Type pored with NaCl				
PCL	2.16 × 10^9^ ± 3.56 × 10^8^	1.55 × 10^7^ ± 5.50 × 10^6^	1.45 × 10^8^ ± 1.14 × 10^6^	PCL and BCP/PCL vs. PCL + Ag and BCP/PCL + Ag *p* < 0.001
BCP/PCL	3.05 × 10^9^ ± 6.65 × 10^8^	2.15 × 10^7^ ± 4.00 × 10^6^	1.24 × 10^8^ ± 1.10 × 10^6^	
PCL + Ag 1%	2.36 × 10^3^ ± 1.88 × 10^2^	4.67 × 10^2^ ± 1.40 × 10^1^	2.43 × 10^2^ ± 1.15 × 10^1^	
PCL + Ag 1.2%	2.01 × 10^3^ ± 6.61 × 10^1^	2.27 × 10^2^ ± 1.83 × 10^1^	2.86 × 10^2^ ± 1.69 × 10^1^	
BCP/PCL + Ag 1%	2.66 × 10^3^ ± 8.54 × 10^1^	3.32 × 10^2^ ± 1.35 × 10^1^	2.52 × 10^2^ ± 1.35 × 10^1^	
BCP/PCL + Ag 1.2%	1.55 × 10^3^ ± 6.53 × 10^1^	2.42 × 10^2^ ± 1.76 × 10^1^	1.42 × 10^2^ ± 1.55 × 10^1^	
B				
Scaffold Type pored with NaNO_3_				
PCL	1.48 × 10^9^ ± 3.80 × 10^8^	2.66 × 10^7^ ± 1.34 × 10^6^	2.19 × 10^8^ ± 1.41 × 10^6^	PCL and BCP/PCL vs. PCL + Ag and BCP/PCL + Ag *p* < 0.001
BCP/PCL	2.82 × 10^9^ ± 9.60 × 10^7^	2.65 × 10^7^ ± 1.89 × 10^6^	1.78 × 10^8^ ± 1.76 × 10^6^	
PCL + Ag 0.79%	3.43 × 10^3^ ± 2.18 × 10^2^	2.30 × 10^2^ ± 1.66 × 10^1^	3.12 × 10^2^ ± 1.83 × 10^1^	
PCL + Ag 1%	2.31 × 10^3^ ± 2.30 × 10^2^	2.01 × 10^2^ ± 3.10 × 10^1^	1.06 × 10^2^ ± 1.28 × 10^1^	
BCP/PCL + Ag 0.79%	6.18 × 10^3^ ± 2.86 × 10^2^	2.52 × 10^2^ ± 1.89 × 10^1^	2.89 × 10^2^ ± 1.50 × 10^1^	
BCP/PCL + Ag 1%	4.96 × 10^3^ ± 1.22 × 10^2^	2.44 × 10^2^ ± 2.17 × 10^1^	1.60 × 10^2^ ± 1.12 × 10^1^	

**Table 5:** Number of planktonic staphylococci and *E. coli* (log_10_ colony-forming units, CFU/mL) in the presence of PCL and BCP/PCL 3D scaffolds, pure or functionalized with low silver concentrations and pored with NaCl (A) or NaNO_3_ (B), towards the three assayed bacterial strains.

	Number of Planktonic Bacteria as log_10_ CFU/mL (Means ± Standard Error of the Means)			Statistical Analysis
A	***S. aureus***	***S. epidermidis***	***E. coli***	**Student’s *t*-Test**
Scaffold Type Pored with NaCl				
PCL	2.80 × 10^9^ ± 2.82 × 10^8^	2.64 × 10^8^ ± 6.62 × 10^6^	1.74 × 10^9^ ± 3.13 × 10^8^	PCL and BCP/PCL vs. PCL + Ag and BCP/PCL + Ag *p* < 0.001
BCP/PCL	2.35 × 10^9^ ± 7.25 × 10^7^	3.09 × 10^8^ ± 1.21 × 10^7^	1.07 × 10^9^ ± 9.35 × 10^7^	
PCL + Ag 1%	1.28 × 10^5^ ± 1.18 × 10^4^	3.32 × 10^4^ ± 1.28 × 10^3^	1.67 × 10^3^ ± 1.14 × 10^2^	
PCL + Ag 1.2%	1.06 × 10^5^ ± 5.53 × 10^3^	2.82 × 10^4^ ± 1.80 × 10^3^	1.30 × 10^3^ ± 1.60 × 10^2^	
BCP/PCL + Ag 1%	1.65 × 10^5^ ± 1.64 × 10^4^	2.80 × 10^4^± 1.55 × 10^3^	3.50 × 10^3^ ± 2.51 × 10^2^	
BCP/PCL + Ag 1.2%	1.58 × 10^5^ ± 2.40 × 10^4^	1.72 × 10^4^ ± 1.39 × 10^3^	2.62 × 10^3^± 1.64 × 10^2^	
B				
Scaffold Type pored with NaNO_3_				
PCL	2.16 × 10^9^ ± 5.81 × 10^8^	2.02 × 10^8^ ± 2.00 × 10^7^	1.42 × 10^9^ ± 1.56 × 10^8^	PCL and BCP/PCL vs. PCL + Ag and BCP/PCL + Ag *p* < 0.001
BCP/PCL	3.67 × 10^9^ ± 3.22 × 10^7^	3.51 × 10^8^ ± 2.75 × 10^7^	1.34 × 10^9^ ± 4.33 × 10^7^	
PCL + Ag 0.79%	2.78 × 10^5^ ± 2.76 × 10^4^	2.79 × 10^4^ ± 2.13 × 10^3^	1.48 × 10^3^ ± 1.07 × 10^2^	
PCL + Ag 1%	1.57 × 10^5^ ± 1.46 × 10^4^	2.61 × 10^4^ ± 2.03 × 10^3^	1.24 × 10^3^ ± 1.33 × 10^2^	
BCP/PCL + Ag 0.79%	3.35 × 10^5^ ± 8.26 × 10^3^	2.76 × 10^4^± 1.02 × 10^3^	2.24 × 10^3^ ± 8.05 × 10^1^	
BCP/PCL + Ag 1%	2.32 × 10^5^ ± 4.21 × 10^4^	2.23 × 10^4^ ± 5.80 × 10^2^	2.06 × 10^3^± 1.00 × 10^2^	

## Data Availability

The source data underlying tables and figures are available from the authors upon request.
